# Impacts of climate change on cropping patterns in a tropical, sub-humid watershed

**DOI:** 10.1371/journal.pone.0192642

**Published:** 2018-03-07

**Authors:** Confidence Duku, Sander J. Zwart, Lars Hein

**Affiliations:** 1 Environmental Systems Analysis Group, Wageningen University, Wageningen, The Netherlands; 2 Africa Rice Center (AfricaRice), Cotonou, Benin; 3 Faculty of Geo-Information Science and Earth Observation (ITC), University of Twente, Enschede, The Netherlands; Louisiana State University College of Agriculture, UNITED STATES

## Abstract

In recent decades, there have been substantial increases in crop production in sub-Saharan Africa (SSA) as a result of higher yields, increased cropping intensity, expansion of irrigated cropping systems, and rainfed cropland expansion. Yet, to date much of the research focus of the impact of climate change on crop production in the coming decades has been on crop yield responses. In this study, we analyse the impact of climate change on the potential for increasing rainfed cropping intensity through sequential cropping and irrigation expansion in central Benin. Our approach combines hydrological modelling and scenario analysis involving two Representative Concentration Pathways (RCPs), two water-use scenarios for the watershed based on the Shared Socioeconomic Pathways (SSPs), and environmental water requirements leading to sustained streamflow. Our analyses show that in Benin, warmer temperatures will severely limit crop production increases achieved through the expansion of sequential cropping. Depending on the climate change scenario, between 50% and 95% of cultivated areas that can currently support sequential cropping or will need to revert to single cropping. The results also show that the irrigation potential of the watershed will be at least halved by mid-century in all scenario combinations. Given the urgent need to increase crop production to meet the demands of a growing population in SSA, our study outlines challenges and the need for planned development that need to be overcome to improve food security in the coming decades.

## Introduction

Increasing crop production in sub-Saharan Africa (SSA) is urgently needed. The population of the region is projected to double by 2050 compared to 2015 [1]. About 97% of current cropland area is under rainfed cultivation [2] and current productivity levels for major food crops, which are the lowest in the world, are inadequate to meet the projected food demand [3]. To meet the food demand of a growing population several options for increasing crop production must be harnessed. These include amongst others crop intensification in rainfed systems to produce higher yields and/or increased cropping frequency, expansion of irrigated area and rainfed cropland expansion. Over the past decades, higher yields, increased cropping frequency (i.e. sequential cropping and intercropping) and cropland expansion have accounted for an estimated 38%, 31% and 31% respectively of the recorded increases in crop production in SSA [3].

In the coming decades, climate change will affect these various options for increasing crop production. West Africa in particular has been identified as a regional hotspot of climate change with climate departure from historical variability projected to occur faster than the global average [4–6]. Changes in precipitation and temperature will pose serious risks to crop production systems and food security in general [7–10]. Several studies have examined the impact of climate change on crop yields in SSA. For example, in Benin, reduced rainfall and increased rainfall variability is likely to result in yield reductions in maize and yam [11] and, in Niger, climate change will reduce millet production between 11% to 26% by 2025 [12]. A variety of mechanisms drive impacts of climate change on crop yields. For example, in Tanzania, climate change is likely to intensify rice diseases such as bacterial leaf blight leading to greater yield losses [13]. Climate change will also affect irrigated agriculture in the Sahel region where, yields of irrigated rice systems are projected to decline by up to 45% by the 2070s [14]. Across SSA, aggregated mean yields for major food crops are forecasted to decrease by 6% to 24% by the end of this century [15]. However, focussing on crop yield responses alone underestimates the impact of climate change on agriculture in SSA. For example, in a key agricultural region in Brazil, analyses of the sensitivities of crop yields, cropping frequency and cropping area to inter-annual climate variability showed that about 70% of the total change in agricultural output in the region could be attributed to changes in cropping frequency and/or cropping area [16]. In SSA, to date, information on the likely impacts of climate change on 1) rainfed cropping frequency in cultivated areas; 2) potential arable land; and 3) the irrigation potential are rare.

In SSA, sequential cropping in rainfed areas has been one of the ways of increasing crop yields (in addition to intercropping) and involves cultivation of two or more crops on the same field after each other or with overlapping growing periods (relay cropping) [17]. Already, sequential cropping in rainfed systems large parts of SSA is constrained by the length of growing period, high labour intensity, lack of knowledge and lack of market access [15]. In the coming decades, detailed information on how climate change will affect sequential cropping in both cultivated areas and in potential arable areas will be vital to reduce agricultural vulnerability to climate change.

In addition to cropping frequency, increased application of irrigation water is vital to boosting crop production and meeting the food demand of a growing population in SSA [2, 18–20]. Investments in irrigation are therefore increasing and irrigation water withdrawals in SSA are expected to increase from 96 km^3^ (2005 estimate) to 133 km^3^ per annum by 2050 [3]. However, changes in precipitation and temperature are not only likely to affect water availability for irrigation but also irrigation water requirements of major food crops in SSA. Furthermore, population growth and socioeconomic development are also likely to increase water demand and hence increase competition for water use. Hence, to support agricultural development and planning, there is a need for detailed information on how climate change and socioeconomic development will jointly affect irrigation potential. Yet, despite numerous studies examining the impact of climate change on water resources in general [21–25], studies specifically examining the impacts on the capacity to support irrigation expansion are rare in SSA.

Therefore, to provide a better understanding of the varied impacts of climate change on opportunities for increasing crop production, we examine three potential options for increasing crop production in a large sub-humid tropical watershed in central Benin, the Upper Ouémé watershed. First, we analyse the impact of climate change on the potential for increasing rainfed cropping frequency through sequential cropping in cultivated areas. Second, we analyse the impact of climate change on the suitability of potential arable land areas for rainfed sequential cropping. Finally, we analyse the combined impacts of climate change and socioeconomic development on the potential for irrigation expansion in the watershed taking into account household water demand and riverine environmental flow requirements, which are the two major non-agricultural water uses in the watershed. Our approach combines hydrological modelling and scenario analysis involving two contrasting Representative Concentration Pathways (RCPs), two water-use scenarios for the watershed based on the Shared Socioeconomic Pathways (SSPs), and environmental water requirements leading to sustained water flows in the Upper Ouémé river network.

## Methods

### Study area

**Fig 1:**
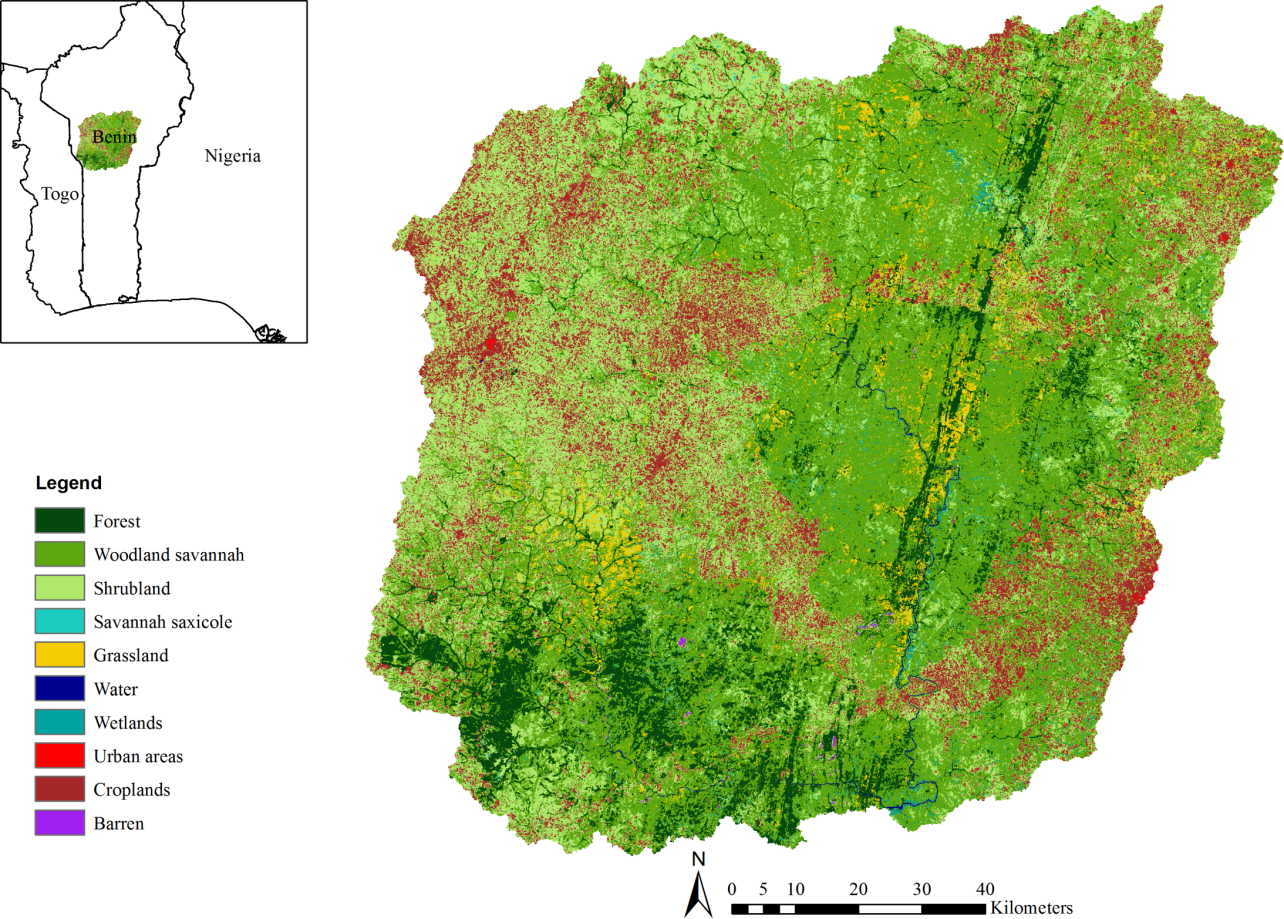
Land cover of the Upper Ouémé watershed showing current cropland areas.

The Upper Ouémé watershed in central Benin covers an area of approximately 14,500 km^2^ with an estimated population of about 510,000 people [26] (Fig 1). It is located in the sub-humid tropical zone and is characterized by a unimodal rainfall season from May to October with about 1250 mm of precipitation per year. In general, Benin is affected by a seasonal alteration of cool and humid monsoon air mass (originating from the Gulf of Guinea), and hot, dry and dusty Saharan air mass [27]. Rainfall anomalies in Benin and West Africa in general have been associated with the northward or southward position of the Inter-Tropical Convergence Zone and the associated low and upper level jet streams [27]. The natural vegetation of the watershed is a mosaic of savannah woodland and small forest islands. Smallholder rainfed agriculture is the major economic activity. Maize, rice, yam, cassava, sorghum and millet are the most important food crops, with cotton being the major cash crop. The cropping intensity of these staple crops is 1.5 [28], indicating that a substantial portion of land either devoted to these crops or other crops is harvested twice per year. The irrigation sector is poorly developed and the lack of irrigation water during the dry season is a major problem for many farmers [29]. Pastoral communities from neighbouring countries such as Nigeria often migrate to this study area, especially for grazing in the dry season when water and food resources are scarce in the less humid zones of the Upper Ouémé to the north [30].

### Modelling the hydrological response of the watershed

To simulate the hydrological response of the watershed under current and future climate conditions, we used a modified Soil and Water Assessment Tool (SWAT), which had been reconfigured with a grid-based landscape discretization [31] and further enhanced to simulate water flow across the discretized landscape units [32, 33]. The SWAT model in general is a spatially explicit, physical, ecohydrological model that simulates the impact of land use and land management practices on water, sediments and agricultural chemicals in large complex watersheds with varying soils, land use and management conditions over several years [34, 35]. The reconfiguration to a grid-based landscape discretization scheme [31, 36] from the standard Hydrological Response Units (HRUs), enhances the spatial detail and accuracy of simulated hydrological processes [32]. Furthermore, a landscape routing sub-model was incorporated to enhance the spatial interaction between discretized landscape units and allowed for the simulation of surface water, lateral and groundwater flow interactions across these units. Detailed information about the grid-based SWAT landscape model description can be found in Rathjens et al. [33] and Arnold et al. [32].

The grid-based SWAT landscape model used in the present study to simulate hydrological response of the watershed under current climatic and future climatic conditions had been set up, calibrated and validated in Duku et al. [37]. The input data used to set up the model are presented in Table 1. The Soil Conservation Service curve number approach was used to model surface runoff and the daily curve number value was calculated as a function of plant evapotranspiration [34] and potential evapotranspiration was modelled with the Hargreaves method [38]. The model was calibrated and validated using daily observed streamflow data from 11 monitoring stations within the watershed (See S1 File) [37]. Calibration was mostly from 2001 to 2007 and validation was from 2008 to 2011. Calibration and validation of the model was carried out using the Sequential Uncertainty Fitting (SUFI-2) optimization algorithm of the SWAT Calibration and Uncertainty Program [39]. See S1 File for the calibrated parameter values (Table A), graphical (Figure A) and statistical results (Table B) of model calibration and validation.

**Table 1:** Description of spatial input data of the Upper Ouémé watershed for the SWAT landscape model.

Data type	Description	Resolution	Source
Topography	ASTER Digital Elevation Model (DEM)	30m	NASA
Land use/ land cover	Classified LANDSAT-7 ETM+ image	28.5m	IMPETUS [30]
Soil types	Soil map and associated parameters derived from geological maps and field surveys	1:200,000	IMPETUS [30]
Precipitation	Gridded daily precipitation data (1999 to 2012)	25km	AMMA-CATCH [40]
Temperature	Gridded monthly average minimum and maximum temperatures (1999 to 2012)	50km	CRU TS 3.21 [41]
Household water consumption	Groundwater and surface water extractions	(village level)	IMPETUS [30]

- NASA is the National Aeronautics and Space Administration of the United States

- IMPETUS is Integrated Approach to Efficient Management of Scarce Water Resources in West Africa

- AMMA-CATCH is the African Monsoon and Multidisciplinary Analysis–Coupling the Tropical Atmosphere and the Hydrological Cycle

- CRU TS is the Climate Research Unit Time Series datasets.

### Climate change and socioeconomic pathways

For the present study, simulations involving daily time-steps were undertaken for the period from 2003 to 2012. This represented the current climatic conditions henceforth referred to as the baseline conditions. To simulate the hydrological response of the watershed under future climate scenarios, climate data (precipitation and temperature) projected under two contrasting Representative Concentration Pathways (RCPs) were utilized. RCPs encompass four greenhouse gas concentration trajectories adopted by the Intergovernmental Panel on Climate Change and supersedes the Special Report on Emissions Scenarios projections [42]. For the present study, precipitation and temperature data projected under RCP2.6 and RCP8.5 for different time-periods were used for SWAT simulation (Table 2). The RCP2.6 scenario is an emission pathway that leads to the lowest concentration levels of atmospheric greenhouse gases [5, 43]. It represents a peak in greenhouse gas emissions by 2050 followed by a consistent decline throughout the rest of this century. It is the pathway needed to realize the targets set during the twenty-first Conference of Parties of the UN Framework Convention on Climate Change, i.e. keep mean global warming to within 2°C above pre-industrial levels. The RCP8.5 scenario, on the other hand, is characterized by increasing greenhouse gas emissions leading to the highest concentration of atmospheric greenhouse gases by the end of this century [5, 43]. It is representative of the business-as-usual scenario, i.e. a continued increase in greenhouse gas emissions.

**Table 2:** Changes in annual precipitation totals and average temperature in future climate scenarios compared to baseline scenario. The average temperature in baseline is 27.4°C.

Scenario and time period	Watershed-wide average annual precipitation total (mm/yr)	Watershed-wide change in average temperature (°C)
Baseline	1240	-
RCP2.6 2040s	1180	0.6
RCP2.6 2090s	1160	0.6
RCP8.5 2040s	1180	1.3
RCP8.5 2090s	1145	3.9

For simulations under climate-change scenarios, we obtained downscaled monthly precipitation totals, and maximum and minimum temperature data projected under RCP2.6 and RCP8.5; and for two time-periods i.e. 2041–2050 (2040s) and 2091–2100 (2090s). Daily precipitation and temperature data are the outputs of multi-model ensemble of 17 General Circulation Models (GCMs) (see S2 File) and were obtained from the MarkSimGCM geoportal (http://gisweb.ciat.cgiar.org/MarkSimGCM/) [44]. The MarkSimGCM geoportal is part of the Consultative Group for International Agricultural Research (CGIAR) research program on Climate Change Agriculture and Food Security (CCAFS). In MarkSimGCM, fifth-order polynomials are fit to climate anomalies between the baseline [45] and each GCM future climate prediction. Cubic convolutions are then used to interpolate to smaller grids of about 1 km resolution. These intra-GCM differences are then added to the baseline data to provide monthly averaged climate data at each grid cell [44]. To produce multi-model ensemble data drawing from all 17 GCMs, the polynomial functions that were fitted to each individual GCM were averaged and not the climate data produced by the GCMs, which would have led to a progressive dilution of the variance as more GCM models were added. A detailed description of the downscaling and bias correction approach can be found in [44].

To generate the daily time-series of climate data needed to run the SWAT model, we used the change factor approach [46, 47, 48]. The change factor approach involves the calculation of the relative changes in monthly precipitation and the absolute changes in temperature between the multi-model GCM data and the baseline climate data for each time period (i.e. 2040s and 2090s) under each RCP scenario [34]. The change factors were calculated separately for each of the 44 subwatersheds of the Upper Ouémé watershed (the SWAT model assigns one climate station to each subwatershed based on the nearest neighbour) [34]. The calculated changes were used to perturb the baseline climate observations in the calibrated and validated grid-based SWAT landscape model. In simulating watershed hydrology under each RCP scenario, the effect of increased atmospheric carbon dioxide concentration was not taken into account. We discuss the implications of excluding increasing atmospheric carbon dioxide in Section 4.

**Fig 2:**
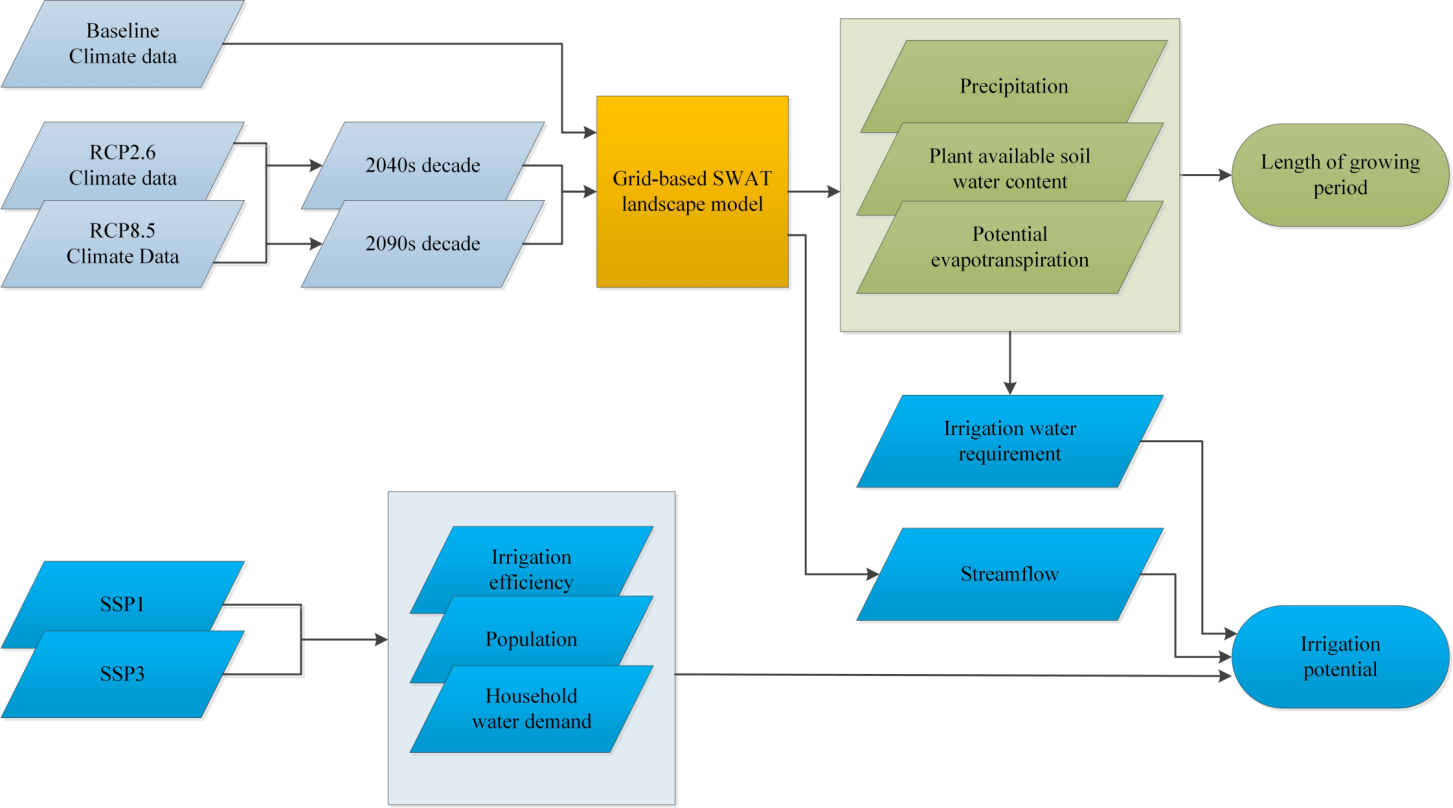
Schematic diagram of the modelling aproach.

In addition to the RCPs, we developed water-use scenarios in line with the qualitative narratives of the Shared Socioeconomic Pathway (SSP) [49, 50] for the calculation of irrigation potentials (Fig 2). The SSPs are a set of alternative reference assumptions about future socioeconomic development in the absence of climate policies. The SSP1 scenario depicts a development pathway characterised by rapid economic development especially in low-income countries leading to rapid technological development, increased resource use efficiency and low population growth whereas SSP3 represents a scenario with slow economic growth, slow technological development, low resource use efficiency and rapid population growth. In this study, each SSP was characterized by three variables; population growth, irrigation efficiency, and per capita domestic water use (Table 3). Estimates of population projections in the watershed under each SSP for different time-periods were computed from Jones et al. [51]. Data on irrigation efficiency was obtained from Hanasaki et al. [52] and had been derived from the qualitative narrative of each SSP. Finally, we derived estimates of per capita domestic water use in our study area based on the qualitative narratives of each SSP and data from FAO [53].

**Table 3:** Characterization of water-use scenarios in line with the qualitative storylines of the Shared Socioeconomic Pathways (SSPs). Current population in the watershed is about 510,000 [26]. Values of irrigation efficiency are based on [52].

	Baseline water-use	2040s		2090s	
		SSP1	SSP3	SSP1	SSP3
Irrigation efficiency	0.45*	0.52	0.45	0.61	0.45
Population growth (%)^**a**^	-	101	156	114	285
Per capita domestic water use (L day^-1^)	19**	60	20	100	30

**a** Percentage increase in population with 2010 as the base year. For the 2040s and 2090s decades, the projected population of the years 2050 and 2100 respectively were used.

* indicates values of irrigation efficiency under baseline conditions obtained from [54].

** indicates per capita domestic water use under baseline conditions obtained from [55, 56]. Per capita domestic water use was derived from [53] and the qualitative storyline of each SSP scenario.

### Modelling meteorological drought

We characterised meteorological drought conditions under each RCP scenario using the Standardized Precipitation Evapotranspiration Index (SPEI) [57]. The SPEI methodology estimates the severity and frequency of meteorological drought by accounting for changes in evapotranspiration demand caused by changes in temperature in addition to precipitation. It is based on a probability distribution fitted to a time-series of the difference between monthly precipitation and potential evapotranspiration (i.e. climatic water balance) aggregated over different time-scales using a moving window (e.g. 3-month, 6-month etc.). This probability distribution is transformed to the cumulative distribution function of the standard normal distribution (with a mean of 0 and standard deviation of 1). In this study, the regional (i.e. average over the entire watershed) monthly precipitation and potential evapotranspiration time-series were used. Potential evapotranspiration was computed using the Hargreaves method [38]. The sequences of climatic water balance in the baseline and for the 2040s and 2090s in each RCP scenario were used to compute the 6-month (seasonal drought) and 12-month (annual drought) SPEIs.

### Modelling length of growing period

The length of growing period (LGP) indicates the potential for rainfed crop production. We modelled the LGP as the number of days in a year in which moisture supply (i.e. the sum of daily precipitation and plant available soil water content) was equal to or exceeded potential evapotranspiration and temperature was above 5°C (e.g. [58]). The basic concept according to the heat unit theory [59, 60] is that plant growth and development will only occur if the temperature exceeds some minimum threshold. For our study area, which is located in tropical lowlands, the year round minimum daily temperature always exceeds 5°C. Hence, the limiting factor in the computation of LGP was moisture supply. The SWAT model was used to simulate daily soil moisture content for the computation of LGP. The computed LGPs were then used together with the criteria in Table 4, to delineate single, relay and double cropping zones under each climate change scenario. The criteria in Table 2 is based on the Agro-Ecological Zone methodology [61]. Sequential cropping zones delineated in this study do not involve the cultivation of wetland rice, which covers less than 1% of the study area.

**Table 4:** Criteria for delineation of potential rainfed sequential cropping zones under rainfed conditions [61]. Sequential cropping zones are disaggregated into relay and double cropping zones.

Cropping zones	Length of growing period (days)	Accumulated temperature above 5°C over the growing period	Accumulated temperature above 10°C over the growing period
Single cropping	≥ 120	-	-
Relay cropping^a^	≥ 200	≥ 3200	≥ 2700
Double cropping^a^	≥ 240	≥ 4000	≥ 3200

a In double cropping zones, there can be cultivation of two or more crops on the same field after each other whereas in relay cropping zones this involves overlapping growing periods between the two crops

### Modelling streamflow drought

Streamflow droughts affect the availability of water for irrigation and other consumptive purposes. We used two approaches to compute streamflow droughts; the Standardised Streamflow Index (SSI) [62, 63] and Severity-Duration-Frequency curves (SDF) [64]. The SSI approach is similar to the SPEI methodology, however, in this case a non-parametric probability distribution was fitted to the time-series of monthly streamflow totals simulated at the watershed outlet [62, 63]. Unlike the SSI approach, the SDF approach involved daily streamflow simulated at the watershed outlet and streamflow droughts occurred when streamflow was below a specified threshold for at least a specific period of time (e.g. [64, 65]). We used the environmental water requirements of the river as the threshold level. The environmental water requirement is critical for sustaining the natural functioning of riverine ecosystems during periods of high flow in the wet season and low flow in the dry season. We defined the environmental water requirements separately for each month as the streamflow value with a 75% exceedance frequency (Q75) [66]. We estimated these threshold levels based on flow duration curves under baseline climatic conditions. For example, the Q75 value of the month of August was 138 m^3^s^-1^ whereas the value in November was 22 m^3^s^-1^. See S3 File for a detailed description of the SDF approach.

### Modelling irrigation potential

We estimated the irrigation potential from a water resources perspective i.e. assuming surface water availability was the major limiting factor [67]. We created a matrix of RCPs and SSPs, where each RCP was characterized by 1) the simulated total streamflow in excess of the Q75 threshold level and 2) potential evapotranspiration (crop water demand); and each SSP was characterized by 1) irrigation efficiency, 2) population growth and 3) per capita domestic water use (Table 3). Irrigation potential (in hectares) was computed as the quotient between total volume of streamflow available and irrigation water requirement [67]. The volume of streamflow available for irrigation was computed using Eq 1. The total irrigation water requirement was computed as the quotient between crop water demand (using rice as a proxy crop) and irrigation efficiency. We used rice as a proxy crop for crop water demand because rice is the most commonly irrigated crop in Benin. Furthermore, land-use data with the required level of detail to distinguish between different crops in croplands are currently unavailable. Obtaining such information is complicated by the small plot sizes and cropping patterns varying from year to year. We discuss the implication of the use of rice as a proxy crop for water demand in Sect. 4,.

$$W=[\sum_{i}\left(V_{t}-Q75\right)]-\left(D\times P\right)$$ (1)

where W is total annual volume of streamflow available for irrigation (m^3^ yr^-1^); V_t_ is total monthly volume of streamflow at month i (m^3^); Q75 is environmental flow requirement of month i (m^3^); D is per capita domestic use (m^3^ person^-1^ yr^-1^); P is total watershed population.

## Results

### Present and projected patterns of meteorological drought

**Fig 3:**
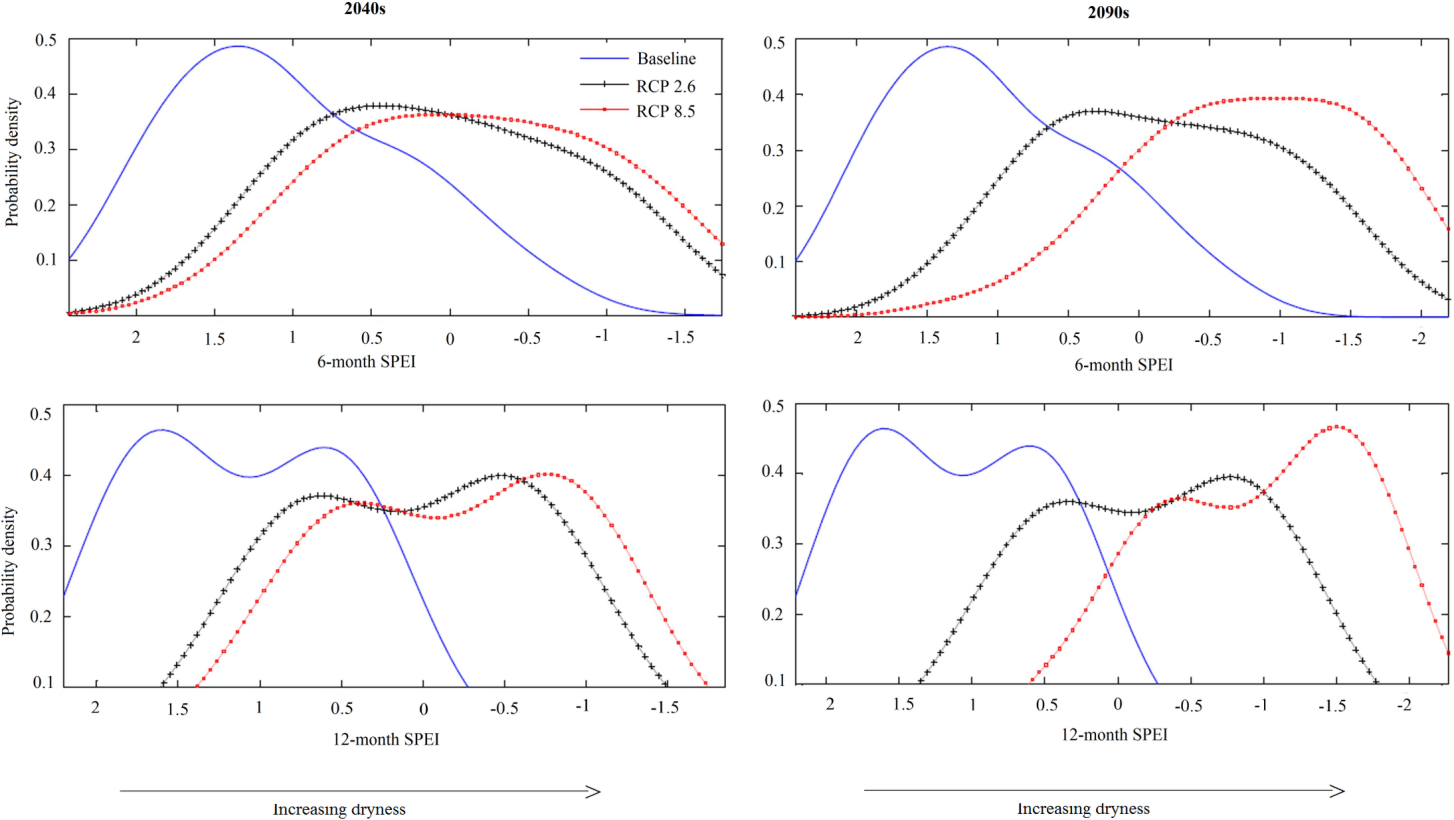
Probability density plots of 6-month and 12-month Standardized Precipitation Evapotranspiration Index (SPEI) derived under baseline climate conditions (2003–2012) and under two future climate scenarios. SPEI values < 0 represent meteorological drought and the greater the absolute value, the higher the severity. SPEI values > 0 represent wetter than normal conditions.

SPEI values are the number of standard deviations by which the anomaly in climatic water balance deviates from the long-term mean. Our SPEI analyses clearly show a substantial shift from relatively wetter climatic conditions under the baseline to increasingly drier climatic conditions under both RCP2.6 and RCP8.5 scenarios (Fig 3). Not only will the probability of occurrence of seasonal drought (6-month SPEI) increase under both RCP2.6 and RCP8.5 but annual droughts (12-month SPEI) will increase as well.

### Impact of climate change on rainfed production potential

**Fig 4:**
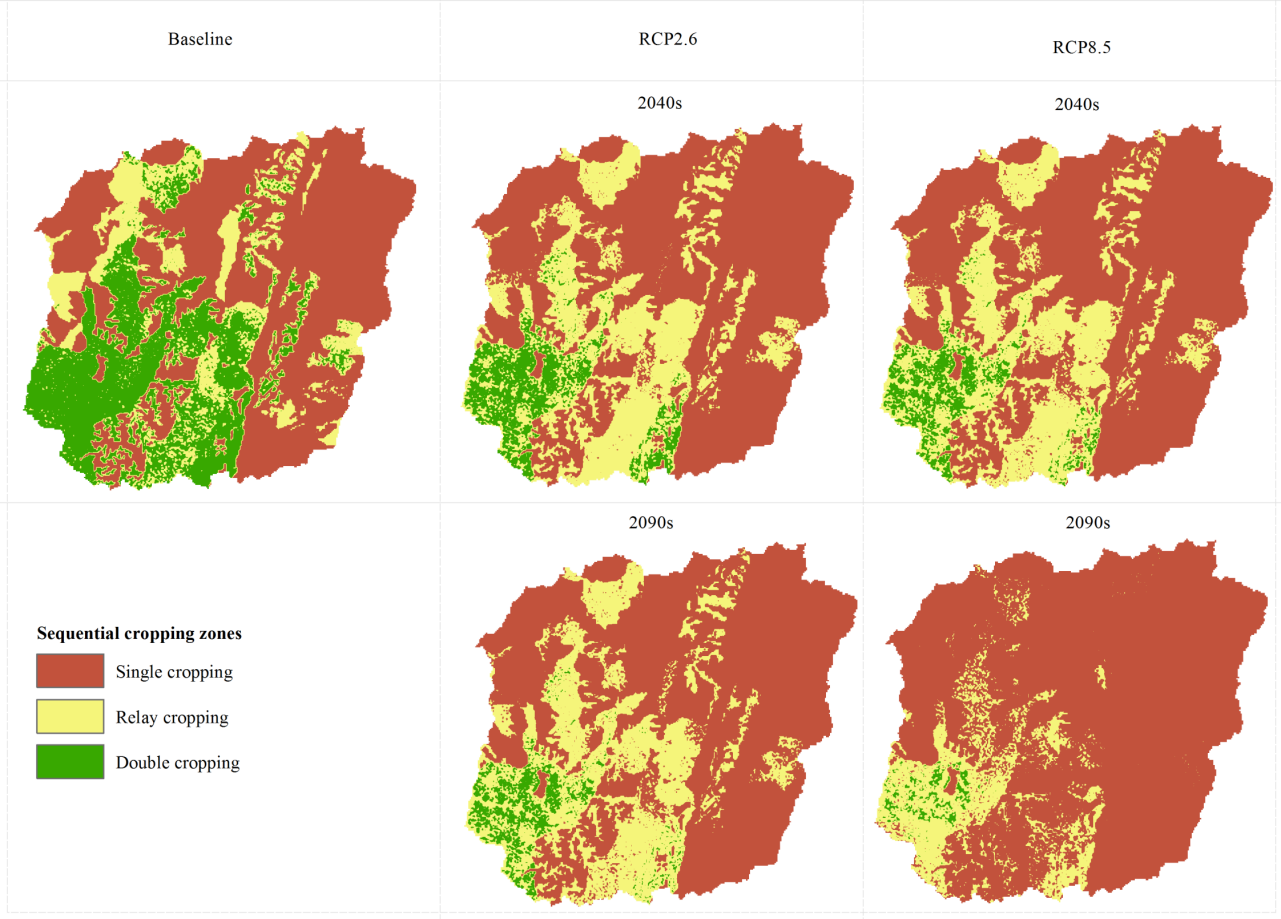
The cropping zones in the watershed under baseline climatic conditions and RCP scenarios. Sequential cropping zones have been disaggregated into full double cropping and relay cropping zones. These zones indicate the areas where water availability is sufficient to permit different cropping systems.

**Fig 5:**
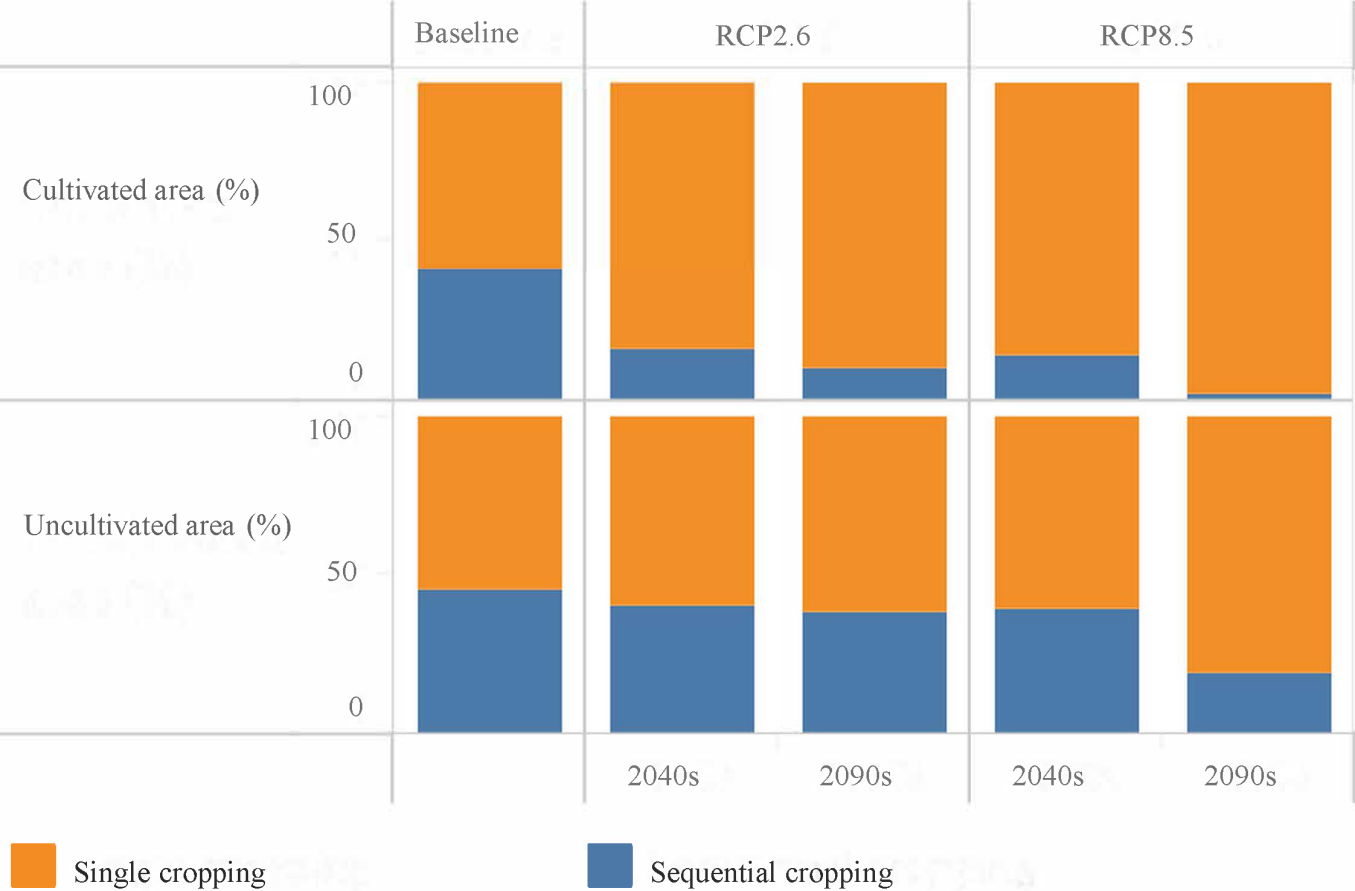
Proportion of cultivated and potential arable areas suitable for sequential and single cropping under different RCP scenarios in the watershed. The total cultivated areas in the watershed are 150,000 ha and the potential arable land area is about 1.3 million ha.

Our analyses show that a watershed-wide average of between 15 and 30 growing days will be lost depending on the climate change scenario. Despite the loss of growing days, cultivated and uncultivated areas that currently are used for single cropping or can support it will still be suitable depending on the type of crop cultivated. However, substantial areas of hitherto rainfed sequential areas will only be suitable for single cropping (Figs 4 and 5). Depending on the climate change scenario, between 50% (30,000 ha) and 95% (57,000 ha) of cultivated areas that are currently used for rainfed sequential cropping or can support it will only be suitable for single cropping (Figs 4 and 5). In currently uncultivated areas, between 10% and 60% of the areas where rainfed sequential cropping is currently feasible will only be suitable for single cropping. Currently over 90% of areas that can support sequential cropping in the watershed, i.e. over 570,000 ha, are not under cultivation and lie mainly in the forested south-western part of the watershed (Fig 4). In the coming decades and especially under RCP2.6, a large part of these areas will still be able to support sequential cropping albeit with either a loss or shortening of the fallow period (Fig 5). Adequate soil and nutrient management will then be required to increase productivity.

### Impact of climate change on streamflow drought

**Fig 6:**
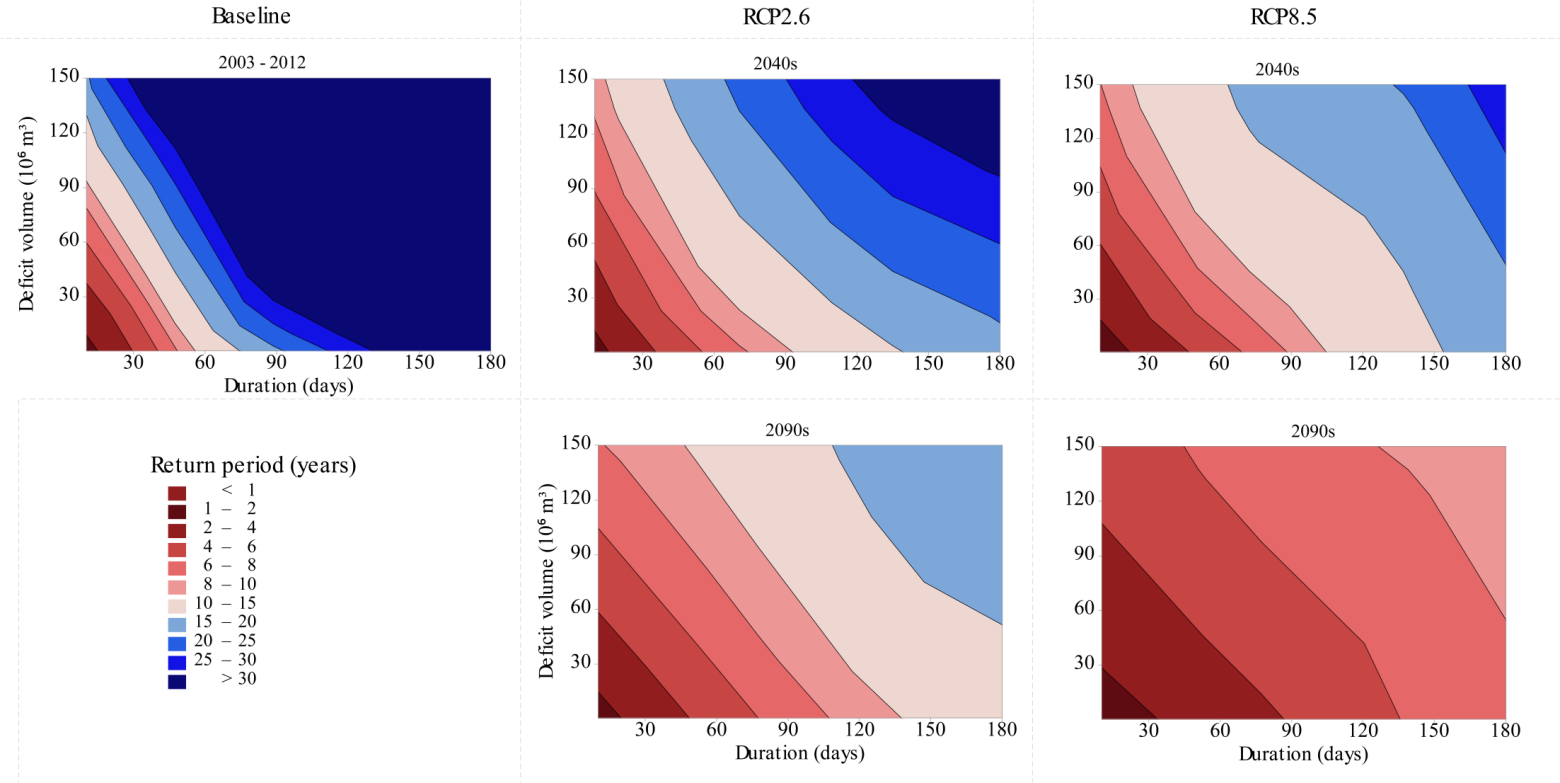
Contour plots showing severity-duration-frequency relationships of streamflow droughts in the Upper Ouémé watershed under baseline climatic conditions (2003–2012) and two RCP scenarios. Contour plots were derived using daily streamflow simulated at the watershed outlet.

**Fig 7:**
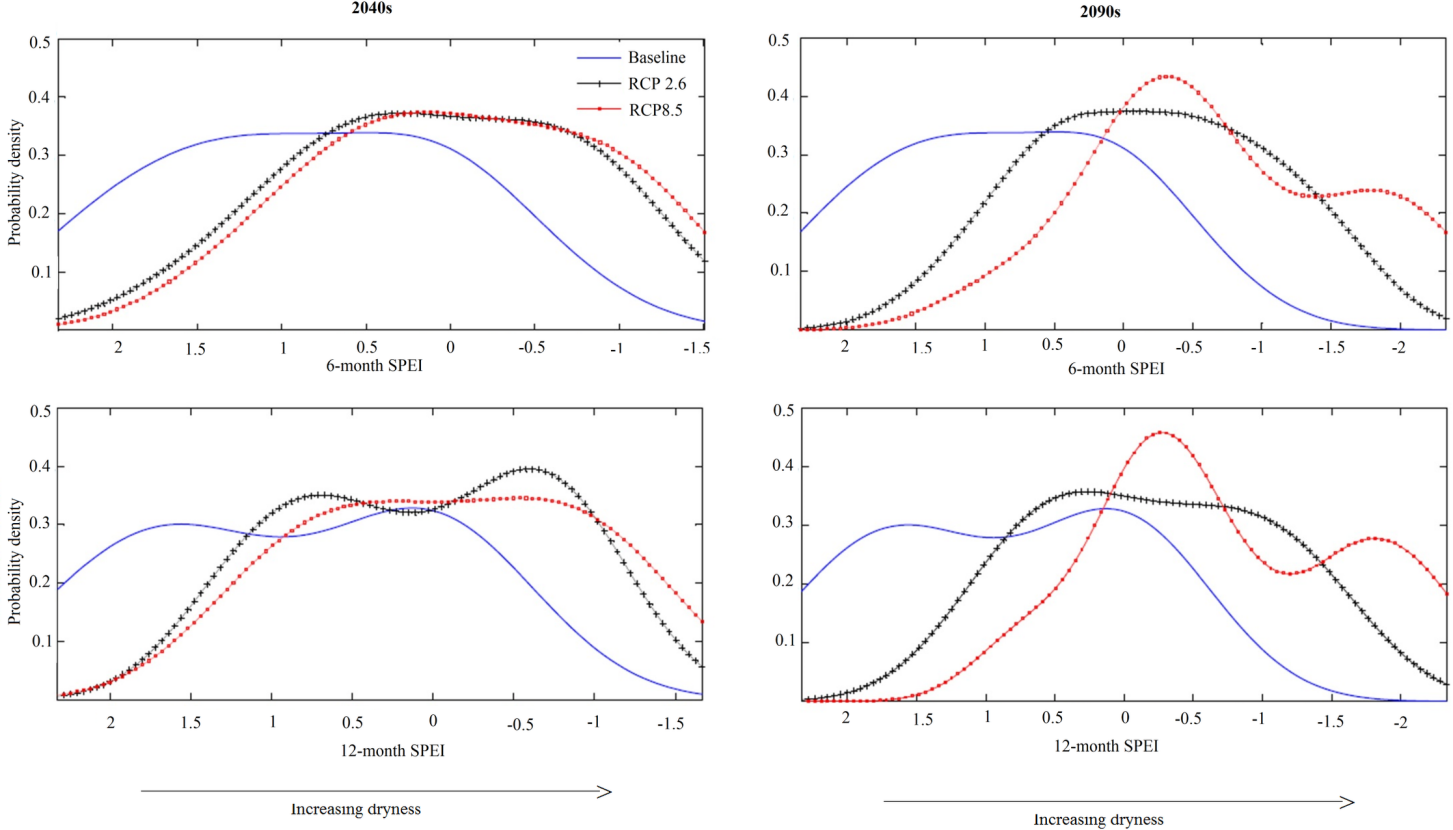
Probability density plots of 6-month and 12-month Standardized Streamflow Index (SSI) derived under baseline climatic conditions and under RCP scenarios. SSI values < 0 represent streamflow drought and the greater the absolute value, the higher the severity. SSI values > 0 represent higher streamflow.

**Fig 8:**
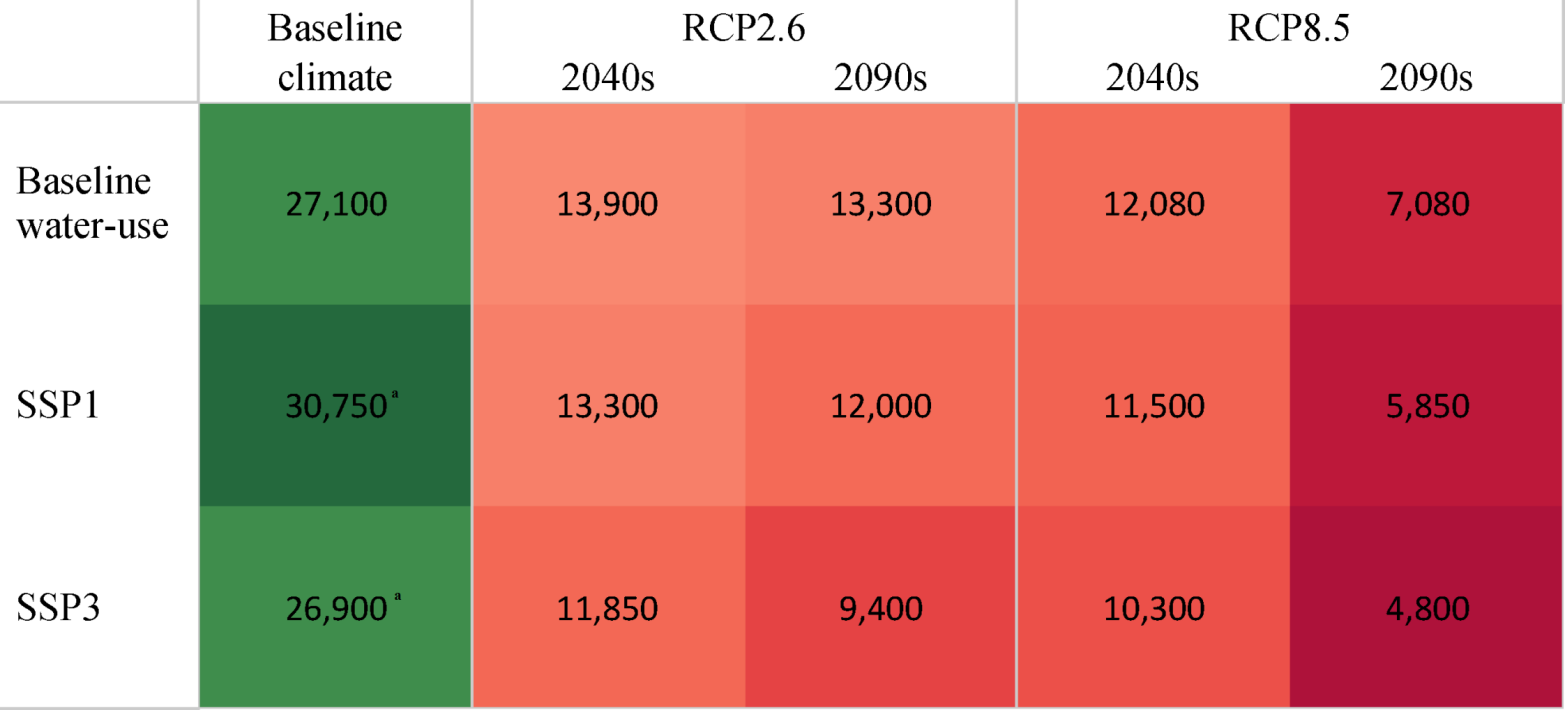
Irrigation potential (ha) in the Upper Ouémé watershed under current and future climate and water-use scenarios. Superscript ^a^ indicates that irrigation potential estimates represent the 2040s under the SSPs and baseline climate. For 2090s, irrigation potential under baseline climate and SSP1 was 36,100 ha whereas it was 26,900 ha under baseline climate and SSP3.

Our SDF and SSI analyses show that climate change will increase the severity, duration and frequency of streamflow droughts (Figs 6 and 7). For example, a streamflow drought event with 120 days duration and a total deficit volume of about 50 million m^3^ water in the watershed is estimated to occur once every 50 years (return period) under baseline climatic conditions. However, in the 2040s, these return periods are projected to be 18 years and 13 years under the RCP 2.6 and RCP 8.5 scenarios respectively (Fig 6). Increasing severity, duration and frequency of streamflow drought affects the availability of water for household consumption, riverine ecosystem requirements and irrigation. In addition to population growth, streamflow droughts will increase competition for water resources (Fig 8). In this study, because we prioritized household water demand and riverine ecosystem requirements over irrigation, streamflow droughts substantially reduce the irrigation potential of the watershed.

### Impact of climate change and socioeconomic development on irrigation potential

Fig 8 shows the irrigation potential of the watershed under different combinations of climate change based on RCPs and socioeconomic development based on SSPs. It shows that the irrigation potential is likely to be at least halved under all combinations of future climatic conditions based on RCPs and socioeconomic development based on SSPs. This can be mainly attributed to reduced precipitation and increased temperature resulting in reduced water availability for irrigation and increased irrigation water requirements. Even though increased irrigation efficiency under SSP1 compared to current conditions may reduce the irrigation intensity, this reduction cannot completely offset the effects of climate change and to a lesser extent population growth.

## Discussion

In this study, we used multi-model ensemble climate data projected by 17 GCMs for simulation. In using multi-model ensemble data, extreme climatic values projected by individual GCMs are smoothed. To maintain the climate extremes, climate data from individual GCMs with divergent climatic trajectories can be used. However, in West Africa, where these projections are highly uncertain especially for precipitation, and there is a large spread across various GCMs [8, 68], using climate data projected by individual GCMs may not address the structural uncertainties and independent simulation errors in these models. In these situations, multi-model ensemble data are highly recommended and have been widely used [69]. The multi-model ensemble approach have been reported to outperform individual ensemble members in hindcasting studies and thus provide an improved ‘best estimate’ forecast [69]. Lambert et al. [70] showed that simulations of multi-model ensemble data of precipitation, temperature, and pressure of current climate are generally closer to observed distributions, as measured by mean squared differences, correlations, and variance ratios, than the results of any particular model. Yira et al. [71] show that as a result of the high uncertainties in precipitation projections in West Africa, a larger ensemble of climate projections is required to estimate the impacts of climate change on water resources accurately. Giorig et al. [72] suggest that as a result of these uncertainties, a minimum of four to five multi-model ensemble is needed to obtain robust regional precipitation change estimates.

We also used the scenario matrix approach [73] to analyse the impacts of climate change based on RCPs and socioeconomic development based on SSPs on the irrigation potential of the watershed. It has been pointed out that the radiative forcing projected under some RCPs may be inconsistent with the socioeconomic assumptions described in some SSPs i.e. certain SSPs may not produce the level of greenhouse gas emissions needed to achieve the level of radiative forcing in certain RCPs. However, such inconsistencies occur only at the global scale of analysis. At the local and regional scales, all possible combinations of RCPs and SSPs may be possible. Because 1) the global-average level of radiative forcing described in each RCP may differ from those at the local and regional scales due to a number of factors, including land-use change and air pollutant concentrations [73]; 2) SSPs are developed as hypothetical cases without new climate policy interventions (mitigation and adaptation) and without being influenced by future climate change [49, 73].

In our analysis, we used the same land-cover and soil parameters for simulation of the watershed hydrology under both current and future climatic conditions. This is one of the main sources of uncertainty in our study. Land-cover dynamics affect actual evapotranspiration and together with soil properties influence the partitioning of rainfall into overland flow and soil infiltration. Incorporating plausible land-use changes could impact on our analyses in a variety of ways and the net effect on streamflow depends on the types and extent of land-use changes. For example, conversion of forests and woodlands for crop cultivation reduces dry season streamflow and consequently water available for irrigation [67]. Nevertheless, by maintaining these watershed characteristics constant, we were able to isolate the impacts of climate change on watershed hydrology from the impacts of plausible land-use changes. We excluded the effects of elevated atmospheric carbon dioxide under future climatic conditions in our analysis, which is another source of uncertainty. The effect of changes in atmospheric carbon dioxide on watershed hydrology is incorporated in the SWAT model through a modification of the Penman-Monteith equation for computing potential evapotranspiration [34]. In this study, however, we used the Hargreaves equation [38] to compute potential evapotranspiration because of inadequate data to apply the Penman-Monteith equation. Elevated atmospheric carbon dioxide has countervailing effects on the transpiration rate of vegetation cover. On one hand, it reduces transpiration rate by reducing leaf stomatal conductance [46, 74, 75]. On the other hand, it increases transpiration rate by stimulating plant growth [46, 75, 76]. Their net effect on the magnitude and seasonality of the components of the hydrological cycle depends on local weather conditions and vegetation characteristics. For example, in forested watersheds of the northern Coastal Ranges and Sierra Nevada mountain range in California, research has shown that elevated carbon dioxide concentrations reduced evapotranspiration by around 3% and consequently increased streamflow [46]. More research, however, is needed on the net effect in the sub-humid tropics of West Africa dominated by woodland savannah.

In this study, we computed irrigation potential from a water resources perspective, i.e. assuming water availability was the only limiting factor for irrigation and all other socioeconomic, biophysical and technological factors were non-limiting. The irrigation potential reported in this study, therefore, is the maximum irrigable land area because, in practice, other physical and socioeconomic factors will be constraining. Our approach reflects the fact that renewable water resources that are adequate for irrigating a given amount of land today may not be so in the future as a result of climate change. Moreover, unlike water availability, many other limitations may be overcome in the future depending on the socioeconomic development pathway. Hence, analysing the impact of climate change on maximum irrigable land area from a water resources perspective is instructive and important for long-term irrigation development planning. Our irrigation potential estimates were computed using rice as a proxy crop for crop water demand. Rice is currently the only staple crop grown on irrigated fields in the watershed. Compared to other crops, rice has a relatively high water demand especially if measured on a per hectare basis (as opposed to a per kg of produce basis). Hence for other crops, our approach underestimates the irrigation potential. Our model can be adapted in a relatively simple way to assess the amount of other crops that can be irrigated.

In rainfed production systems that are characteristic of the watershed and SSA in general, crop growth and yield are closely related to the LGP. Across SSA, inadequate LGP is one of the major constraints to establishing rainfed sequential cropping systems. In this study, we have demonstrated that increasing drought risk due to climate change will considerably reduce the LGP across the study area. Due to the reduction in LGP, substantial areas in both cultivated and uncultivated areas hitherto suitable for rainfed sequential cropping will revert to single cropping. Cultivated and uncultivated areas that can currently support single cropping will still be suitable in the coming decades. However, the number of crops that can be cultivated (especially long-cycle crops such as cassava and yam) will be limited as a result of the shortening of the LGP. Our analyses show that currently about 60,000ha (40%) of cultivated areas in the study area are suitable for sequential cropping (including relay cropping). It is difficult to ascertain if all of these areas are actually used for sequential cropping due to inadequate data. However, the average cropping intensity of staple crops such as maize, yam, cassava and sorghum in the study area is 1.5, i.e. the harvested area is one and a half times greater than the physical area devoted to the cultivation of each crop [28]. This can be attributed to both sequential cropping and intercropping. If all 60,000ha of suitable sequential cropping areas in cultivated areas are currently used for sequential cropping, then in the coming decades, farmers will have to shift to either single cropping systems or adopt crop cultivars with shorter growing periods. To a degree, the impact on crop production may be mitigated by agronomy including breeding of drought resistant varieties. If, however, a substantial portion is only used for single cropping, then climate change will severely limit such opportunities for increasing crop production. In both situations, substantial reductions in sequential cropping areas may result in relatively greater rainfed cropland expansion to make up for lost opportunities to increase crop production.

Over 90% of the total suitable sequential cropping area is currently not under cultivation and lies in the forested south-western part of the watershed. In these areas, the higher soil water-holding capacity allows for relatively longer LGPs and the impact of climate change is relatively less. However, most of these potentially suitable sequential cropping areas are presently either forested or are woodland savannahs. The forested areas are essential for biodiversity conservation, wood resources, water flow regulation, carbon sequestration etc. and the woodland savannah areas provide grazing opportunities for livestock. Particularly during the dry season, pastoral communities from other parts of Benin and neighbouring countries, such as Nigeria, often migrate here for grazing [30]. Nevertheless, it is likely that forested and woodland savannah areas will be increasingly under pressure from land use change in the coming decades. Among others, this may cause tensions between pastoralists traditionally using the areas for grazing and new settlers. Land use changes in the Upper Ouémé watershed are also likely to affect population growth and per capita water use. These feedbacks are, however, not included in the present study and it would be very challenging to do so in the scope of one paper.

In Benin, the total actual land area equipped for irrigation is only 23,000ha [77]. In our study area, irrigation is almost non-existent. The lack of irrigation water during the dry season has been a major problem for many farmers [18, 29]. To sufficiently increase crop production in the coming decades, irrigation will have to play a crucial role and is a pathway that has been proposed for other parts of SSA [20, 78]. Under current climatic conditions, there is considerable potential for irrigation expansion. A maximum land area of 27,000ha can be irrigated even after household water demands and environmental water requirements have been implemented. However, our study has shown that future opportunities for irrigation expansion will be heavily constrained by increased severity, frequency and duration of streamflow droughts. Streamflow droughts coupled with increased household water-use due to population growth and socioeconomic development will result in increased competition for surface-water resources. Where household and environmental water requirements are prioritized over irrigation as in this study, streamflow droughts will substantially reduce the irrigation potential of the watershed. Potential cropland expansion into currently forested areas will also affect seasonal distribution of streamflow further reducing irrigation opportunities especially in the critical dry season [67]. Deforestation tends to increase peak flow in the wet season and reduce baseflow in the dry season.

## Conclusion

In this study, we have shown that, in addition to crop yield responses, climate change will affect other options that have been used to increase crop production in recent decades in SSA i.e. rainfed sequential cropping, rainfed cropland expansion and irrigation expansion. Currently, about 41% of cultivated areas in the Upper Ouémé watershed are either used for rainfed sequential cropping or can support it. However, by 2050 this will decrease to between 2% and 16% depending on the climate change scenario. Farmers will therefore have to shift to single cropping systems or adopt improved agronomic practices including drought-resistant and short-cycle cultivars. Farmers may also be driven to expand to hitherto uncultivated areas to make up for lost opportunities to increase crop production. In the Upper Ouémé watershed, over 90% of the land area that can support rainfed cropping is not currently under cultivation and largely consists of forest and woodland savannah. This situation is unlike other parts of Benin where the availability of currently uncultivated land is much lower. A large part of these potential arable lands will still be able to support rainfed sequential cropping in the coming decades despite the loss of between 15 and 30 growing days due to their relatively higher soil moisture storage. If these areas are to be used for rainfed sequential cropping, then fallow periods will have to be shortened or lost completely and improved soil and nutrient management will be needed to increase productivity. However, in a previous paper, Duku et al. [67], we showed that the conversion of forested and woodland savannah areas to cropland will have negative feedbacks on water availability for irrigation. In the present study, we have shown that even if there is no change in forest cover, at least 50% of irrigation potential will be lost in the coming decades due to climate change. Forest and woodland areas, therefore, will be needed to regulate water flows and increase dry season streamflow in addition to the provision of other ecosystem services. Our paper shows the importance of using an integrated approach to rural development planning, where climate change can be expected to have multiple, major implications on cropping systems and resilience for climate change depends upon maintaining overall landscape integrity including areas that regulate water flows.

## Supporting information

S1 File Supporting information document contains Table B (Calibrated parameter values of the SWAT landscape model); and Table B and Figure A (SWAT calibration and validation results).
(DOCX)

S2 File Supporting information document contains Table A listing the general circulation models used in the study.
(DOCX)

S3 File Supporting information document contains severity-duration-frequency methodology for modelling streamflow droughts and Table D which shows the goodness of fit of marginal distributions.
(DOCX)
