# Supplementary material for: Impacts of climate change on cropping patterns in a tropical, sub-humid watershed
Source: PLoS One. 2018 Mar 7;13(3):e0192642. doi: 10.1371/journal.pone.0192642 (PMC5841656; doi:10.1371/journal.pone.0192642)
Supplement: S1 File — Supporting information document contains Table B (Calibrated parameter values of the SWAT landscape model); and Table B and Figure A (SWAT calibration and validation results). (DOCX) [file pone.0192642.s001.docx]

**S1: SWAT model calibration and validation results**

**Table A.** Calibrated parameter values of the SWAT Landscape model. Superscript **a** indicates that the fitted values depended on the land cover type. Superscript **b** indicates that this parameter was used only in the calibration of the grid-based SWAT Landscape model. Subscript **v_** indicates that the parameter value is replaced by the fitted value. Subscript **r_** indicates the parameter value is multiplied by (1 + the fitted value).

| Parameter name | **Description** | **Fitted values** |
| --- | --- | --- |
| _r__CN2 | Initial SCS runoff curve number for moisture condition II | (from -0.2 to -0.05)^a^ |
| _v__RCHRG_DP | Deep aquifer percolation fraction | 0.2 |
| _v__GW_REVAP | Groundwater re-evaporation coefficient | 0.18 |
| _v__GWQMN | Threshold depth of water in the shallow aquifer required for return flow to occur | 1000 |
| _v__REVAPMN | Threshold depth of water in the shallow aquifer for re-evaporation or percolation to the deep aquifer to occur | 500 |
| _v__SURLAG | Surface runoff lag coefficient | 0.12 |
| _r__SOL_AWC | Available water capacity of the soil | 0.1 |
| _v__ESCO | Soil evaporation compensation factor | (from 0.001 to 0.2)^a^ |
| _v__EPCO | Plant uptake compensation factor | (from 0.1 to 1)^a^ |
| _v__USLE_P | USLE equation support practice factor | 0.13 |
| _v__USLE_C | Minimum value of USLE C factor for water erosion applicable to the land cover | (from 0.038 to 0.45)^a^ |
| _v__NPERCO | Nitrate percolation coefficient | 0.2 |
| _v__N_UPDIS | Nitrogen uptake distribution parameter | 70 |
| _v__SDNCO | Denitrification threshold water content | 1.1 |
| _v__CDN | Denitrification exponential rate coefficient | 1.4 |
| _v__DD^b^ | Drainage density factor which affects the flow separation ratio | 7.5 |

**Fig A.** Comparing simulated and observed streamflow data for three monitoring stations.

**Table B.** Calibration and validation results for streamflow. NSE is Nash-Sutcliffe efficiency, PBIAS is percent bias, and RSR is ratio of the root mean square error to the standard deviation of measured data.

| Monitoring stations | Drainage area (km^2^) | Calibration | | | Validation | | |
| --- | --- | --- | --- | --- | --- | --- | --- |
|  |  | **NSE** | **PBIAS** | **RSR** | **NSE** | **PBIAS** | **RSR** |
| Upstream stations |  |  |  |  |  |  |  |
| Affon-Pont | 1 172 | 0.69 | 27.0 | 0.56 | 0.62 | 15.9 | 0.62 |
| Aval-Sani | 760 | 0.70 | 12.0 | 0.55 | 0.64 | 7.8 | 0.60 |
| Bori | 1 608 | 0.65 | -24.7 | 0.59 | -0.49 | -121.4 | 1.22 |
| Tebou | 522 | 0.47 | 43.5 | 0.72 | 0.58 | 20.3 | 0.65 |
| Downstream stations |  |  |  |  |  |  |  |
| Beterou | 10 046 | 0.85 | 5.7 | 0.39 | 0.78 | -17.8 | 0.47 |
| Barerou | 2 128 | 0.71 | 20.8 | 0.54 | 0.72 | -22.7 | 0.53 |
| Cote-238 | 3 040 | 0.69 | 3.5 | 0.56 | 0.68 | -18.4 | 0.56 |
| Igbomakoro | 2 309 | 0.76 | 11.3 | 0.49 | 0.71 | -4.0 | 0.54 |
| Sarmanga | 1 334 | 0.48 | 23.2 | 0.72 | 0.44 | 17.2 | 0.75 |
| Aguimo | 394 | 0.25 | -20.9 | 0.87 | 0.12 | -.60.1 | 0.94 |
| Wewe | 297 | 0.42 | 21.6 | 0.76 | 0.42 | -6.5 | 0.76 |
