# Supplementary material for: Impacts of climate change on cropping patterns in a tropical, sub-humid watershed
Source: PLoS One. 2018 Mar 7;13(3):e0192642. doi: 10.1371/journal.pone.0192642 (PMC5841656; doi:10.1371/journal.pone.0192642)
Supplement: S2 File — Supporting information document contains Table A listing the general circulation models used in the study. (DOCX) [file pone.0192642.s002.docx]

**S2: General Circulation Models**

**Table C.** General Circulation Models used in this study

| **General Circulation Model** | **Institution** |
| --- | --- |
| BCC-CSM 1.1 | Beijing Climate Center, China Meteorological Administration |
| BCC-CSM 1.1(m) | Beijing Climate Center, China Meteorological Administration |
| CSIRO-Mk3.6.0 | Commonwealth Scientific and Industrial Research Organisation and the Queensland Climate Change Centre of Excellence |
| FIO-ESM | The First Institute of Oceanography, SOA, China |
| GFDL-CM3 | Geophysical Fluid Dynamics Laboratory |
| GFDL-ESM2G | Geophysical Fluid Dynamics Laboratory |
| GFDL-ESM2M | Geophysical Fluid Dynamics Laboratory |
| GISS-E2-H | NASA Goddard Institute for Space Studies |
| GISS-E2-R | NASA Goddard Institute for Space Studies |
| HadGEM2-ES | Met Office Hadley Centre |
| IPSL-CM5A-LR | Institut Pierre-Simon Laplace |
| IPSL-CM5A-MR | Institut Pierre-Simon Laplace |
| MIROC-ESM | Atmosphere and Ocean Research Institute (The University of Tokyo), National Institute for Environmental Studies, and Japan Agency for Marine-Earth Science and Technology |
| MIROC-ESM-CHEM | Atmosphere and Ocean Research Institute (The University of Tokyo), National Institute for Environmental Studies, and Japan Agency for Marine-Earth Science and Technology |
| MIROC5 | Japan Agency for Marine-Earth Science and Technology, Atmosphere and Ocean Research Institute (The University of Tokyo), and National Institute for Environmental Studies |
| MRI-CGCM3 | Meteorological Research Institute |
| NorESM1-M | Norwegian Climate Centre |
