# Supplementary material for: Impacts of climate change on cropping patterns in a tropical, sub-humid watershed
Source: PLoS One. 2018 Mar 7;13(3):e0192642. doi: 10.1371/journal.pone.0192642 (PMC5841656; doi:10.1371/journal.pone.0192642)
Supplement: S3 File — Supporting information document contains severity-duration-frequency methodology for modelling streamflow droughts and Table D which shows the goodness of fit of marginal distributions. (DOCX) [file pone.0192642.s003.docx]

**S3: Severity-Duration-Frequency method for modelling streamflow drought**

Using the monthly variable threshold approach, the seasonality of streamflow is preserved and there is an inherent recognition that streamflow droughts (or negative streamflow anomalies) could occur even during wet seasons. In other words, we assumed streamflow was not available for potential consumptive uses below the environmental water requirement. Using the daily streamflow time-series simulated under baseline and RCP scenarios, streamflow drought events were identified based on two criteria. First, a streamflow drought event must be made up of at least seven days of streamflow below a threshold level [1]. Second, two separate drought events should occur at least five days apart or they were considered as a single drought event. The sequences of identified drought duration and drought severity were each fitted to a range of marginal probability distributions (e.g. Logistic, Normal, Generalized Extreme Value, Gamma etc.). The goodness of fit of each marginal distribution was evaluated based on the negative log likelihood test, Bayesian Information Criterion and Akaike Information Critierion (Table D). Based on these tests, the time-series of duration were fitted to the Generalized Extreme Value distribution whereas the associated severity series were fitted to a lognormal distribution.

To model the bivariate joint cumulative distribution of duration and severity of streamflow drought events, we used copulas [2]. Droughts are complex, stochastic phenomena with multiple correlated attributes for which multivariate methods provides a more complete characterization. However, applying traditional multivariate frequency analysis methods is unsuitable because drought attributes may require different distribution functions [4, 5] and may be correlated. Copula functions, hence provide a suitable approach for characterizing drought characteristics and to derive the joint distribution of dependent drought attributes [6]. Over the last decade, copulas have emerged as a method for addressing multivariate problems in several disciplines including hydro-climatology [7]. The use of copula functions allowed for the quantification and the relation of severity and duration of streamflow droughts to their frequency of occurrence under different climate change scenarios. This approach can be applied in large areas of SSA where water resource planning and allocation, including irrigation planning, design and management requires a detailed knowledge of streamflow reliability. Copulas are a type of functions that join univariate distribution functions to form multivariate distribution functions. We fitted the marginal distributions to a series of copula functions (Gumbel, Frank, Clayton and Gaussian). The Akaike Information Criterion (AIC) [8, 9] was used to evaluate the goodness of fit of each copula. The AIC is a measure of the relative quality of statistical models for a given dataset. Based on this test, the Gaussian copula was the most suitable and was used to model the joint cumulative distribution function of duration and severity of streamflow droughts. One hundred random sequences of streamflow drought events characterised by severity and duration were generated with the fitted copula function and Eq. (A) was used to compute the joint return periods.

$T_{r}= \frac{1}{P (S\geq s, and D\geq d)}$

$= \frac{1}{1- F_{S}\left( s \right)- F_{D}\left( d \right)+C[F_{S}\left( s \right), F_{D}\left( d \right)]} (A)$

where *T_r_* is frequency (years); *P* is probability; *S* is the random variable severity of steamflow drought event, *s* is any value of *S (m^3^)*; *D* is the random variable duration of streamflow drought event; *d* is any value of *D*; F_V_(v) and F_D_(d) are the univariate cumulative distribution functions (CDFs) severity and duration respectively; C[F_S_(s), F_D_(d)] is the copula function for computing the joint CDF.

**Table D:** Results of negative log likelihood goodness of fit marginal distributions to streamflow drought duration and severity. The smaller the value, the better the fit.

| **Distribution** | **Baseline** | **RCP2.6 (2040s)** | **RCP8.5 (2040s)** | **RCP2.6 (2090s)** | **RCP8.5 (2090s)** |
| --- | --- | --- | --- | --- | --- |
| **Streamflow drought duration** | | | | | |
| **Generalized Extreme Value** | 111 | 123.7 | 144.7 | 139.1 | 117.4 |
| **Inverse Gaussian** | 113.5 | 131.4 | 148.1 | 145.6 | 117.5 |
| **Loglogistic** | 114.3 | 133 | 151 | 147.9 | 120 |
| **Lognormal** | 114.4 | 134 | 151.5 | 148.3 | 119 |
| **Weibull** | 119.2 | 140.8 | 155.5 | 154.3 | 120.9 |
| **Gamma** | 118.5 | 143.3 | 156.7 | 155.5 | 121.5 |
| **Exponential** | 119.4 | 146.3 | 158.3 | 156.5 | 122.9 |
| **Streamflow drought severity** | | | | | |
| **Generalized Extreme Value** | 489.3 | 522.3 | 557.7 | 561.5 | 411.3 |
| **Inverse Gaussian** | 490.3 | 529.5 | 563.1 | 566.4 | 416.3 |
| **Loglogistic** | 488.9 | 520.2 | 559.7 | 560.6 | 410.3 |
| **Lognormal** | 487.9 | 520 | 558.3 | 559.6 | 409.8 |
| **Weibull** | 488 | 520.9 | 558.8 | 560.2 | 410 |
| **Gamma** | 489.4 | 523.9 | 560.3 | 562 | 411.4 |
| **Exponential** | 497.9 | 541.1 | 582.2 | 581.2 | 423.7 |

**References**

[1] Tallaksen L M and van Lanen H A J 2004 Hydrological drought: processes and estimation method for streamflow and groundwater *Developments in water science* **48**

[2] Sklar K 1959 Fonctions de repartition a n Dimensions et Leura Marges *Publ. Inst. Stat. Univ. Paris* **8** 229-31

[3] Shiau J T and Modarres R 2009 Copula-based drought severity-duration-frequency analysis in Iran *Meteorological Applications* **16** 481-9

[4] Shiau J T 2006 Fitting drought duration and severity with two-dimensional copulas *Water Resources Management* **20** 795-815

[5] Song S B and Singh V P 2010 Frequency analysis of droughts using the Plackett copula and parameter estimation by genetic algorithm *Stochastic Environmental Research and Risk Assessment* **24** 783-805

[6] Mishra A K and Singh V P 2010 A review of drought concepts *Journal of Hydrology* **391** 204-16

[7] Salvadori G, De Michele C, Kottegoda N T and Rosso R 2007 *Extremes in nature: an approach using copulas* vol 56: Springer Science & Business Media)

[8] Akaike H 1974 A new look at the statistical model identification *IEEE Transactions on Automatic Control* **19** 716-23

[9] Fang Y, Madsen L and Liu L 2014 Comparison of two methods to check copula fitting *IAENG International Journal of Applied Mathematics* **44** 53-61
